# Comprehensive analysis of draft genomes of two closely related pseudomonas syringae phylogroup 2b strains infecting mono- and dicotyledon host plants

**DOI:** 10.1186/s12864-016-3358-y

**Published:** 2016-12-28

**Authors:** Rinat I. Sultanov, Georgij P. Arapidi, Svetlana V. Vinogradova, Vadim M. Govorun, Duglas G. Luster, Alexander N. Ignatov

**Affiliations:** 1 Moscow Institute of Physics and Technology (State University), Moscow, Russia; 2 Shemyakin-Ovchinnikov Institute of Bioorganic Chemistry, the Russian Academy of Sciences, Moscow, Russia; 3 Research Center of Biotechnology, Moscow, Russia; 4 SRCC of Physical-Chemical Medicine, Moscow, Russia; 5 USDA-ARS Foreign Disease - Weed Science Research Unit, Ft. Detrick, Washington, DC USA; 6 Russian Peoples’ Friendship University, Moscow, Russia; 7 R&D Center “PhytoEngineering” LLC, Moscow region, Russia

**Keywords:** *Pseudomonas syringae*, Dicots, Monocots, Pan-genome, Core genome, T3SS, Virulence factors

## Abstract

**Background:**

In recent years, the damage caused by bacterial pathogens to major crops has been increasing worldwide. *Pseudomonas syringae* is a widespread bacterial species that infects almost all major crops. Different *P. syringae* strains use a wide range of biochemical mechanisms, including phytotoxins and effectors of the type III and type IV secretion systems, which determine the specific nature of the pathogen virulence.

**Results:**

Strains 1845 (isolated from dicots) and 2507 (isolated from monocots) were selected for sequencing because they specialize on different groups of plants. We compared virulence factors in these and other available genomes of phylogroup 2 to find genes responsible for the specialization of bacteria. We showed that strain 1845 belongs to the clonal group that has been infecting monocots in Russia and USA for a long time (at least 50 years). Strain 1845 has relatively recently changed its host plant to dicots.

**Conclusions:**

The results obtained by comparing the strain 1845 genome with the genomes of bacteria infecting monocots can help to identify the genes that define specific nature of the virulence of *P. syringae* strains.

**Electronic supplementary material:**

The online version of this article (doi:10.1186/s12864-016-3358-y) contains supplementary material, which is available to authorized users.

## Background

In recent years, the damage caused by bacterial pathogens to major crops has been increasing worldwide. *Pseudomonas syringae* [1] is a widespread bacterial species that infects plants and causes many different diseases: leaf and fruit spots, cankers, blights, etc. *P. syringae* is a Gram-negative gamma-proteobacterium that can be isolated from more than 180 host plant species of different taxonomic groups, including nearly all major agricultural crops [2, 3]. Besides being a phytopathogen, this bacterium also occurs as an epiphyte on healthy plants or as a symbiont in phytophagous insects [4]. It is also present in all phases of the natural water cycle: in clouds, rain water, snow, Antarctic ice, and-in association with algae-in streams and rivers. *P. syringae* is one of the most important objects for studying the molecular mechanisms of pathogenesis and plant response to infection [5].

The *P. syringae* species is divided into more than 50 pathovars indistinguishable by their physiological (microbiological) characteristics but infecting specific host plants or causing specific symptoms of a disease. Although P*. syringae* can infect a broad spectrum of plants, its individual strains are only virulent on a limited number of plant species; in the other plants, however, they either induce an immune response or cannot cause a disease at all [6]. Based on the results of DNA–DNA hybridization, Gardan et. al. divided the *P. syringae* species into nine genotypes (genomospecies) [7]. Taxonomic studies performed by multilocus sequence typing (MLST) revealed that the *P. syringae* species can be classified into 7 to 12 phylogenetic groups; some authors merge the *P. cichorii* and *P. viridiflava* with the *P. syringae* species [8–11]. The phylogenetic groups generally correspond to the genomospecies previously determined by DNA–DNA hybridization; this classification is confirmed by the genome-wide analysis of representatives of the species [12].

Different *P. syringae* strains use a wide range of biochemical mechanisms that determine their virulence to specific plants. These mechanisms include phytotoxins, ice-nucleation proteins [13], and effectors of the type III and type IV secretion systems, which determine the specific nature of the pathogen virulence [14]. Bacteria of different *P. syringae* pathovars produce four main phytotoxins: coronatine, phaseolotoxin, syringomycin, and tabtoxin [13]. Though these toxins are potentially important for the bacterial virulence, none of them is sufficient to cause a disease [13, 15].

To date, the genetic diversity of the *P. syringae* population in the Russian Federation remains poorly studied. There are several studies devoted to the comparative evaluation of 16S-23S rRNA intergenic spacer regions in *P. syringae* и *P. fluorescens* and to the rep-PCR analysis of a limited number of *Pseudomonas* strains isolated from cereal crops [16, 17]. Using MLST, Ignatov et. al. (in press, available at 2016) studied a collection of the *P. syringae* strains isolated from different crops and demonstrated that the strains of phylogroup 2 are predominant in Russia (more than 56% of all studied strains isolated from legumes, sunflower, cereal crops, brassicas, cucurbits, and grapes belong to phylogroup 2).

Phylogroup 2 of *P. syringae* comprises the majority of the strains isolated from the most different habitats. It comprises the pathovars *P. s. pv aceris, P. s. pv aptata, P. s. pv atrofaciens, P. s. pv. avellanae, P. s. pv. coryli, P. s. pv dysoxyli, P. s. pv japonica, P. s. pv lapsa, P. s. pv papulans, P. s. pv pisi, P. s. pv solidagae,* and *P. s. syringae*; it also comprises three previously described genetic subgroups (clades) 2a, 2b, and 2c [18]. Subgroup 2b includes typical strains of such widespread phytopathogens as *P. syringae* pv. *syringae*, *P. s. pv. aptata,* and *P. s. pv. atrofaciens*, whereas nonpathogenic *P. syringae* strains, which are phenotypically similar to *P. viridiflava*, belong to subgroup 2c. In general, the strains of phylogroup 2 are characterized by the highest frequency of hypersensitivity reactions (HR) in tobacco plants, diseases in sprouts of wheat and sunflower, and the synthesis of syringotoxin [11].

To assess genetic features of the Russian *P. syringae* strains, we sequenced two *P. syringae* strains, 1845 и 2507, that infect dicots and monocots, respectively. Using phylogenetic analysis, we demonstrated that these strains are related and that they belong to phylogroup 2, clade 2b (according to Berge [11]) together with strains SM and B64, which infect wheat. Our aim was to compare the virulence factors in these and other available genomes of phylogroup 2 to identify genes responsible for the specialization of bacteria.

## Methods

### Strains and pathogenicity tests

Strains 1845 and 2507 were isolated in the Laboratory of Plant Bacterial Diseases at the Russian Research Institute for Phytopathology (Bolshie Vyazemy, Moscow region). The strains were stored at –80 °C in King’s B liquid medium [19] with 15% glycerol. Before use, a culture was replated on King’s B agar medium.

Strain 1845 was obtained from diseased leaves of a sunflower (cv. “Eklor”) collected in the Republic of North Ossetia-Alania in 2010. The strain was highly virulent to different dicots and inhibited the germination of their seeds. Strain 2507 was isolated from a winter wheat plant (cv. “Moscovskaya 39”) with symptoms of leaf blight collected in Krasnodar Krai in 2012. The strain was highly aggressive to monocots.

The pathogenicity and virulence of bacteria were assessed by the ability of the bacteria to induce the hypersensitivity reaction within 12 h after infiltrating leaves of tobacco (*Nicotiana tabacum*, Samsung cultivar) and pelargonium (*Pelargonium × hortorum*) with bacterial suspension. The virulence of bacteria was assessed using a number of dicot and monocot species (Additional file 1). Three methods of plants inoculation were used: (1) excised cotyledon assay-cotyledons were cut off using a scalpel dipped in a suspension of two pathogen concentrations 10^8^ CFU/mL and 10^6^ CFU/mL; (2) bacterial suspension (10^8^ CFU/mL and 10^6^ CFU/mL) was atomized into a true leaf of a plant; and (3) a hundred of seeds were soaked in a bacterial suspension (10^8^ CFU/mL and 10^6^ CFU/mL) for 1 h before their germination on filter paper. In all the inoculation methods, we used bacterial suspensions obtained from 48-h bacterial cultures on King’s B agar medium and diluted with Potassium Phosphate Buffer pH 7.4, 10 mM to the desirable concentrations.

### Phenotypic and biochemical analysis

Morphological, physiological, and biochemical characteristics of bacterial cultures were determined by the methods for the phenotypic differentiation of the *Pseudomonas* genus: LOPAT (described in the manual for the identification of pathogenic bacteria [19]); GATTa tests (gelatin liquefaction, aesculin hydrolysis, tyrosinase activity, and L-tartrate utilization), and the analysis for the ice nucleation activity performed as described in [20].

### DNA extraction and genome sequencing, assembly, and annotation

Bacteria were cultured on King’s B agar medium [19, 20]. Total DNA preparations were isolated from fresh cultures after 2–3 days of growth using the method of the DNA adsorption on magnetic particles (Miniprep kit, LLC Silex, Russia) according to the manufacturer’s instructions.

We sequenced 4–5 μg DNA of strains 1845 and 2507 on a 454 GS-FLX Titanium platform. The obtained readings were assembled into contigs using the GS De Novo Assembler software developed by Roche (http://www.454.com/products/analysis-software/). The sequence was annotated using the RAST Server [21].

### Selection of strains for the joint analysis; phylogenetic analysis and calculation of the average nucleotide identity (ANI) values

To determine the *P. syringae* phylogroup membership, we used the phylogeny by the *citrate synthase* (*cts*) gene. It has previously been shown that the sequence of this gene is sufficiently informative to classify *P. syringae* strains by clades and phylogroups [11]. For the analysis, we selected 87 *P. syringae* strains (Additional file 2) covering all the phylogroups [11]. The tree was constructed by the maximum likelihood method using the RAxML package [22]. *Pseudomonas rhizosphaerae* 6B4 was used as an outgroup.

To infer phylogenetic relationships between the strains of phylogroup 2, we selected 20 *P. syringae* strains with fully sequenced genomes, including 1845 and 2507 (Additional file 2). We then used multilocus sequence typing (MLST) to construct a phylogenetic tree based on the data on 7 household genes (*RNA polymerase sigma factor* – *rpoD*, *Citrate synthase* – *gltA*, *Glyceraldehyde-3-phosphate dehydrogenase* – *gap1*, *DNA gyrase subunit* – *gyrB*, *potassium uptake protein* – *kup, Aconitate hydratase B* – *acnB*, and *Glucose-6-phosphate isomerase* – *pgi*) [23]. Strain DC3000 was used as an outgroup [24]. As it has previously been shown, this method provides a sufficiently accurate reconstruction of the phylogenetic relationships in the *Pseudomonas* species [12].

For 12 strains of phylogroup 2, including 2507 and 1845 (Additional file 2), we also calculated the average nucleotide identity (ANI) between the genomes using the JSpecies package [25].

### Identification of the pan- and core genomes and unique genes; COG-analysis

The entire pan- and core genomes were identified by ortholog search using the blastp software [26]. Genes were considered orthologous if the E-value was less than 1 × 10^−10^, the length of the aligned region was more than 60% of the entire gene length, and the identity of the aligned regions was more than 60%. To identify the entire pan- and core genomes, we selected 20 *P. syringae* strains with fully sequenced genomes, including 1845 and 2507 (Additional file 2). Among the selected strains, 8 strains infect monocots (hereinafter, group M) and 11 strains infect dicots (hereinafter, group D). Strain 1845 was used to identify pan- and core genomes but was not included into the group D because this strain, as we show in this study, has relatively recently changed the class of its host and may introduce errors into the study.

To construct the pan genome of the strains infecting monocots (Additional file 2), the genes found in the group M strains were selected from the entire pan-genome. To construct the pan-genome of the strains infecting dicots, the genes found in the group D strains were selected from the entire pan-genome (Additional file 2). Core genomes were obtained from the respective pan-genomes.

Clusters of Orthologous Groups (COG) analysis was conducted using the WebMGA service [27]. COG class enrichment was calculated using Fisher’s exact test with the fdr correction for multiple testing (*p*-value < 0.05). The search for associations between the COG groups and the host plant class was performed using Fisher’s exact test with the fdr correction for multiple testing.

### Finding the effectors of the type III secretion system (T3SS); T3SS, T4SS, and T6SS gene clusters; phytotoxin genes; quorum sensing genes; and mobile and CRISPR elements

We used the database on the effectors of the type III secretion system in *P. syringae* available on the site http://www.pseudomonas-syringae.org/. Its current version (04.10.2014) contains 128 unique effectors. The search for effectors was performed by the tblastn algorithm [26] with the E-value threshold of 1 × 10^−5^. If the alignment was incomplete, we used Baltrus’s algorithm: we searched for stop codons that are the nearest from the 5′ and 3′ termini of the aligned region. If a start codon is located between the 5′ stop codon and the start of the alignment and if this start codon belongs to the same reading frame as the aligned region and the 3′ stop codon, it is an active effector. Otherwise, the effector is truncated [28]. The search for the clusters of the type III, IV, and VI secretion systems was conducted using the T346Hunter server [29].

To find the genes responsible for the phytotoxin synthesis, we used the blastn algorithm [26] with the E-value cutoff of 1 × 10^−10^ and the identity of 80%. To find the genes responsible for the quorum sensing, we used the blastn algorithm with the E-value threshold of 1 × 10^−10^ and the identity of 80%. Mobile elements were identified by the IS Finder service [30] with the E-value threshold of 1 × 10^−70^. Associations were found using Fisher’s test with the fdr correction for multiple testing (*p*-value < 0.05). CRISPR (Clustered Regularly Interspaced Short Palindromic Repeats) elements were identified using the CRISPR-finder server [31].

## Results

### Characteristics of the strains

Main morphological, cultural, and biochemical properties of strains 1845 and 2507 of the *P. syringae* species are given in Additional file 3. Both strains belong to the LOPAT 1a group [19] and demonstrate a positive reaction for levan production (L); negative reaction for oxidase production (O); negative test result for pectinolytic activity on potato (P); negative reaction for arginine dihydrolase production (A); and negative reactions in the tobacco hypersensitivity test (T) on *Nicotiana tabacum* plants, ‘Samsung’ cultivar, and in the hypersensitivity test on *Pelargonium × hortorum* (pelargonium).

Within 12 h after the infiltration, strains 1845 and 2507 applied in the concentrations of 10^8^ CFU/mL and 10^6^ CFU/mL induced the hypersensitivity reaction in plants.

Moreover, they tested positive for gelatin liquefaction (G) and aesculin hydrolysis (A) and tested negative for tyrosinase activity (T) and tartrate utilization (Ta).

The results of the artificial inoculation of plants with *Pseudomonas syringae* strains 1845 and 2507 are given in Additional file 1.

Strains 1845 used in the concentrations of 10^8^ CFU/mL and 10^6^ CFU/mL induced water-soaked lesions within 48 h after inoculating the leaves of dicots by atomizer. The leaves of monocots did not show such symptoms.

When used in the concentration of 10^6^ CFU/mL, strain 2507 did not induce severe disease symptoms in dicots but did affect monocots. At the concentration of 10^8^ CFU/mL, the symptoms of strain 2507 infection manifested in dicots faster than in monocots and resembled the hypersensitivity reaction.

The inoculation of germinating dicots with strain 1845 used in the concentrations of 10^8^ CFU/mL and 10^6^ CFU/mL decreased the number of germinated seeds and inhibited the growth of seminal roots. No such symptoms were observed when inoculating the seeds of monocots. The inoculation of the plant seeds with strain 2507 slowed down the germination of monocots but did not affect the growth and development of dicots when the concentrations of 10^8^ CFU/mL or 10^6^ CFU/mL were used.

### Sequencing, de novo assembly, and annotation of strains 1845 and 2507

Sequencing produced 145 000 and 135 000 single-end readings with an average length of 716 and 701 for strains 1845 and 2507, respectively. De novo assembly generated draft genomes of strains 1845 and 2507. The length of these genomes was 5.77 and 5.95 Mb; they consisted of 91 and 97 contigs, the lengths of which were more than 500 bp and the N50 of which were 136 and 131 Kb, respectively (Table 1). When annotating, we identified 5207 and 5415 genes for strain 1845 and 2507, respectively, and 5145 and 5358 of these genes were protein-coding (Table 1).

**Table 1:** Key parameters of the assembly and annotation of the genomes of *P. syringae* strains 1845 и 2507

Parameter	Strain 1845	Strain 2507
Genome length (Mb)	5.77	5.95
Number of contigs	91	97
N50 (Kb)	136	131
GC composition (%)	59.22	59.1
Total number of genes	5207	5415
CDS	5145	5358
rRNA (5S, 16S, 23S)	1, 1, 1	1, 1, 1
tRNA	57	53

### Phylogenetic analysis

**Fig. 1:**
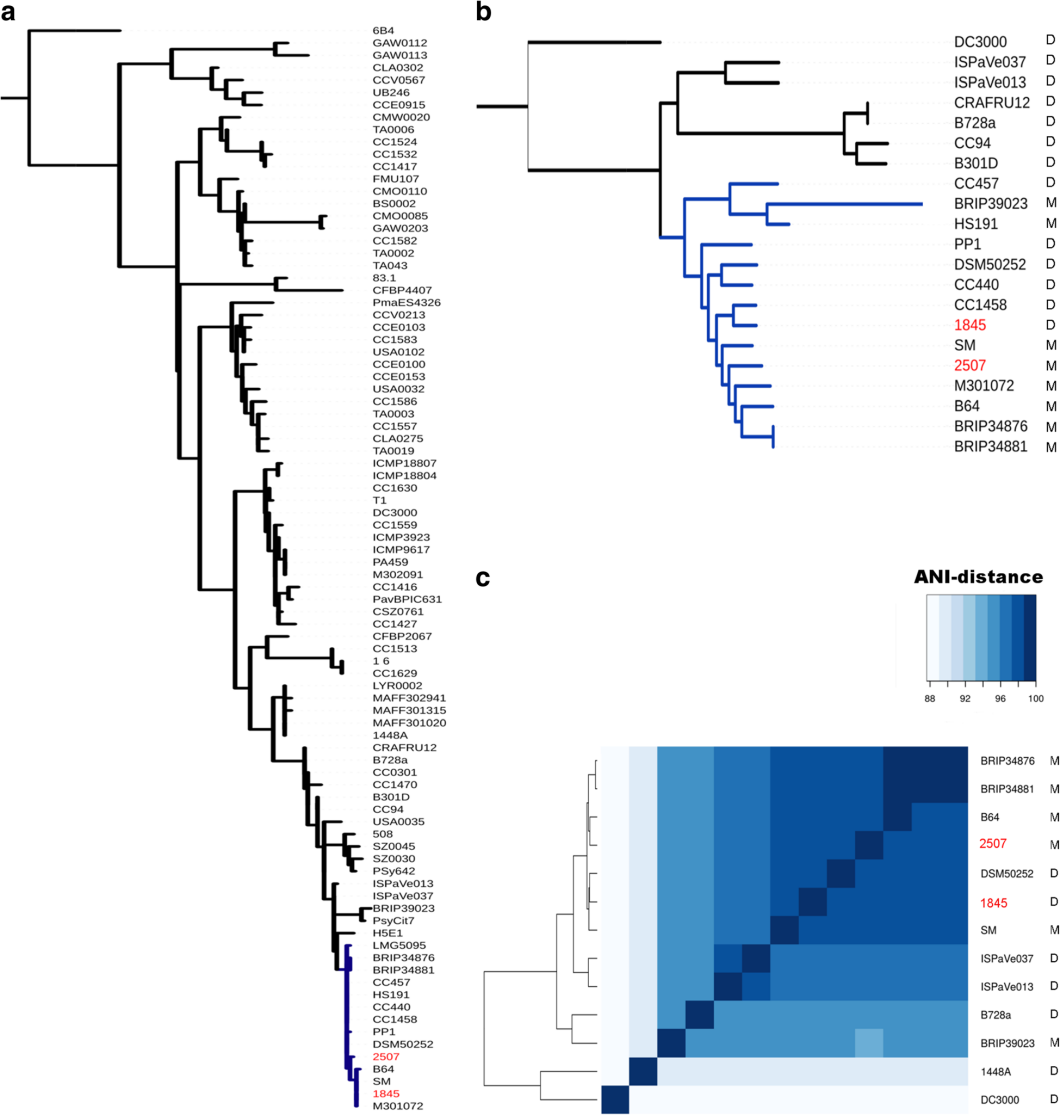
Phylogenetic analysis of strains 1845 and 2507. Russian strains are shown in red. Letters M or D indicate if a strain infects monocots or dicots, respectively. **a** Phylogenetic tree based on the *cts* gene of *P. syringae* 87 strains rooted on *P. rhizophaerae* strain 6B4. The phylogenetic tree is constructed by the maximum likelihood method using the RAxML package [22]. Clade 2b is shown in blue. **b**. Phylogenetic tree for 20 strains of phylogroup 2 rooted on strain DC3000. The phylogenetic tree is based on seven genes (*rpoD*, *gltA*, *gap1*, *gyrB*, *kup*, *acnB*, and *pgi*) with strong bootstrap support. Clade 2b is shown in blue. **c** Clusterization of the strains of phylogroup 2 based on the ANI values

According to the study performed by Berge et. al. on 216 strains, the *P. syringae* species divides into 23 clades distributed by 13 phylogroups, each of which is characterized by its own level of pathogenicity and cold resistance and by the contents of T3SE and mobile elements [11]. To assign the Russian strains to a certain phylogroup, we conducted the phylogenetic analysis of 87 *P. syringae* strains based on the partial *cts* gene sequence using a strain of the *P. rhizosphae* species as an outgroup (Fig. 1a). As it has previously been shown, the tree constructed on the basis of the *cts* gene adequately reflects the distribution of the strains by clades and phylogroups [11]. Based on the resulting tree, we can conclude that the Russian strains belong to phylogroup 2.

To clarify the relationship between the strains, out of the total of 87 strains, we selected 20 strains satisfying the following conditions: all selected strains belong to phylogroup 2, their genomes are fully sequenced, and the classes of their host plants are known (Additional file 2). The *P. syringae* pv. *tomato* strain DC3000 was used as an outgroup [24]. A phylogenetic tree (Fig. 1b) was constructed for these strains based on the MLSA of seven household genes (*rpoD, gltA, gap1, gyrB, kup, acnB,* and *pgi*). The Russian strains, 1845 and 2507, belong to the same clade, namely clade 2b, as the strains B64 and SM isolated from wheat.

To confirm the correct clustering of the strains, we calculated pairwise ANI values between the genomes and performed the clustering based on these values (Fig. 1c, Additional file 4). This clustering confirmed that the Russian strains belong to clade 2b. Moreover, it showed that strains 1845, 2507, SM, and B64 cluster together.

Since strains 1845, 2507, and SM belong to the same clade (2b), we additionally compared their genomes. The contigs of genomes 1845 and 2507 were ordered according to the genome of strain SM and aligned by this genome using the progressive Mauve software [32]. Additional file 5 shows that the genomes of the Russian strains are highly collinear with the SM genome. Long insertions and deletions in the alignment generally correspond to the regions of contig breaks. In strain SM, the chromosome region between PssSM_2181 and PssSM_2256 (57.3 Kb) is a prophage cluster. In strain 2507, the respective region changed its location and orientation (Additional file 6). Strain 1845 lacks this cluster altogether.

Another key indicator of the relationship between strains is the conservation of the hrp-cluster structure. We aligned the hrp clusters of 1845 and 2507 by strain SM (Additional file 7) [29]. The figure shows that the structure of the cluster is conserved between the studied strains.

### Analysis of the pan- and core genomes of phylogroup 2

To analyze the pan- and core genomes of phylogroup 2, we selected 20 strains with fully sequenced genomes, including two Russian strains (Additional file 2). These 20 selected strains were further divided into groups: the group M comprised 8 strains infecting monocots and the group D comprised 11 strains infecting dicots. We did not include strain 1845 into the group D because this strain has relatively recently changed the class of its host and, therefore, may contain both the genes inherent to monocot-infecting strains and the genes inherent to dicot-infecting strains.

The pan-genome of 20 strains comprises 6525 unique genes, whereas their core genome comprises 3171 unique genes (Table 2, Additional file 8).

**Table 2:** Number of genes in the core and pan-genomes of P. syringae phylogroup 2, groups M (monocots) and D (dicots)

	pan-genome	core genome
Second phylogroup (M + D, except strain 1845; 20 strains)	6525	3171
Group M (8 strains)	4647	3371
Group D (11 strains)	5164	3370
Unique genes of the group M	869	199
Unique genes of the group D	1386	198

We annotated the pan-genome of all the 20 strains by COG-classes: 3633 out of the total of 6525 proteins in the pan genome were annotated. The COG-class enrichment was analyzed for the pan- and core genomes of groups M and D using the Fisher’s exact test with the correction for multiple testing. We found no significant differences between the pan- and core genomes of groups M and D (Additional files 9A and 4B). We compared the COG-class enrichment for unique genes of the pan-and core genomes of groups M and D (Additional files 9C and 4D). For the unique genes of the pan-genomes, there is a significant difference in the enrichment of three classes (D, K, and U). For the unique genes of the core genomes, there is a significant difference in the enrichment of seven classes (I, M, N, O, P, Q, and S) (Additional file 10).

For each COG-group, we checked for the random distribution between the strains of groups M and D (Fisher’s test, *p*-value < 0.01). As the result, we found seven COG-groups associated with the host plant class but none of these groups passed the threshold after the fdr correction for multiple testing. All seven groups are present in most of the group D strains (Table 3), and three of the seven groups are present in strain 1845 (YP_234265.1, YP_234264.1, and YP_237386.1).

**Table 3:** COG-groups associated with the ability to infect dicots

COG-group	Description of cog-group	COG-class	Frequency in the group D	Frequency in the group M	*p*-value
YP_235010.1	Transcriptional regulator	K	1	0.125	0.000159
YP_234265.1	Putative threonine efflux protein	E	1	0.125	0.000159
YP_234264.1	Transcriptional regulators	K	1	0.125	0.000159
YP_234628.1	Uncharacterized membrane-associated protein	S	1	0.250	0.001032
YP_237386.1	Transcriptional regulator	K	0.818182	0.125	0.005477
YP_235613.1	Transcriptional regulator	K	0.909091	0.250	0.006271
YP_235987.1	Transcriptional regulator	K	0.909091	0.250	0.006271

### Secretory systems of strains SM, 1845, and 2507 and toxins of the strains of P. syringae phylogroup 2

We analyzed the presence of the genes of three secretory systems participating in pathogenesis (T3SS, T4SS, and T6SS) in strains SM, 2507, and 1845 (Table 4).

**Table 4:** Number of genes of the main secretory systems in *P. syringae* strains 1845, 2507 and SM. Field notation: total number of the system genes in the strain/number of the system core genes in the strain/total number of the core genes in the system

System	Strain 1845	Strain 2507	Strain SM
T3SS 1	15/9/9	15/9/9	15/9/9
T3SS 2	41/11/11	41/10/11	40/10/11
T4SS	4/4/24	4/4/24	12/12/24
T6SS 1	15/11/13	14/11/13	14/11/13
T6SS2	16/10/13	14/9/13	15/10/13

T6SS was initially described in the *Vibro cholera* bacterium [33]. This system was later found in almost a quarter of the species of gram-negative bacteria, mostly in known pathogens. This system is responsible for the transport of the effector proteins participating in the establishment of parasitic or symbiotic relationships between prokaryotes and eukaryotes and for the competition between prokaryotes [34]. T6SS is generally represented by two gene clusters: T6SS-1 and T6SS-2. These two clusters were found in the Russian strains, but the core genes of these clusters are only partially present, which makes the full functioning of T6SS improbable (Table 4).

T4SS is involved in transporting proteins and DNA; particularly, it enables horizontal gene transfer between bacteria or between bacteria and plants [35, 36]. The genes of this system were found in all the three strains. SM strain comprises only 12 out of the total of 24 core components of the system. The Russian strains lost almost all T4SS. Apparently, T4SS is inessential for the virulence of these strains.

The main function of T3SS is the delivery of effector proteins into the cell body of a host [37–39]. In all the studied strains, there is a fully functional cluster T3SS-1 comprised of 15 genes and all of the 9 key genes are present. In the Russian strains, the cluster T3SS-2 only comprises 41 genes, but strain 2507 also lacks one of the 11 key genes of T3SS, whereas all of its components are present in strain 1845. The missing gene is *fliP* (flagellar biosynthesis protein), one of the nine mandatory membrane components [40]. In strain SM, the cluster consists of 40 genes but it also lacks 1 of the 11 key genes of T3SS (Table 4).

**Fig. 2:**
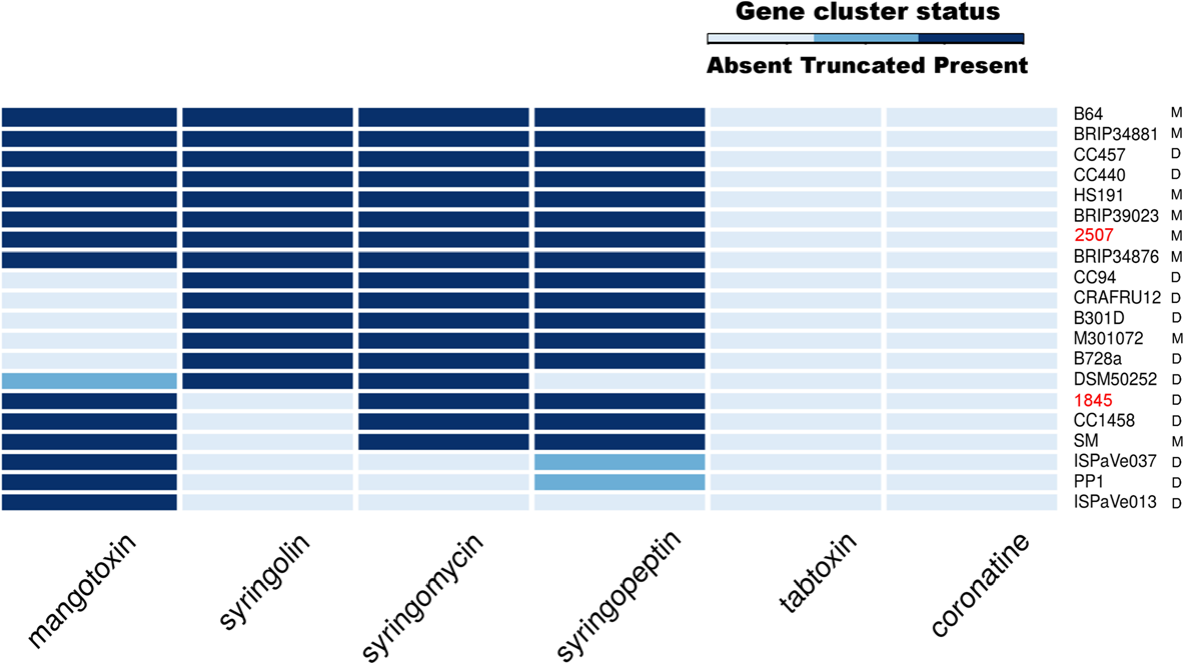
Distribution of the gene clusters responsible for the phytotoxin synthesis among the strains of phylogroup 2. Dark blue indicates the presence of a cluster, blue indicates the absence of some genes in the cluster, and white indicates the absence of cluster. Russian strains are shown in red. Letters M or D indicate if a strain infects monocots or dicots, respectively

All genomes of the phylogroup 2 strains were studied to establish the presence of the gene clusters responsible for the synthesis of six phytotoxins of pseudomonads: mangotoxin, syringolin, syringopeptin, syringomycin, tabtoxin, and coronatine (Fig. 2). It has previously been shown that tabtoxin and coronatine are not typical of the strains of phylogroup 2 [28, 41]. Syringomycin and syringopeptin are cyclic lipopeptide toxins synthesized by the classic NRPS-mechanism [42]. Both of these toxins are present in strains 1845, 2507, and SM. Mangotoxin is an inhibitor of ornithine N-acetyltransferase; it is widespread among the *P. syringae* strains and the apparatus for its synthesis consists of six proteins combined in the mbo-operon [43]. This operon is present in both Russian strains and in strain SM. Syringolin is absent in strains 1845 and SM; it is present, however, in strain 2507.

### T3SS effector repertoire of phylogroup 2 and the Russian strains

**Fig. 3:**
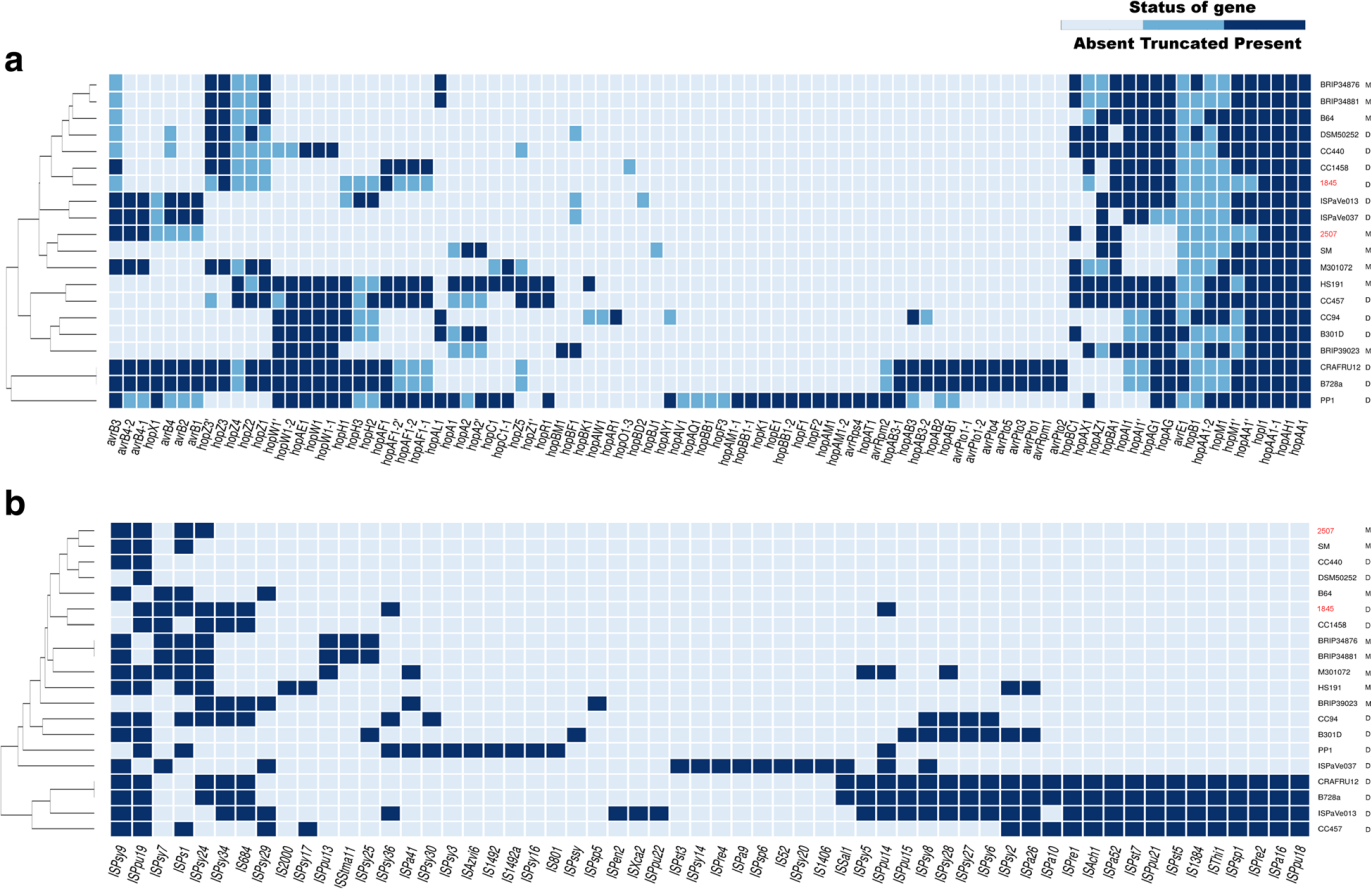
Distribution of genes of effectors of the type III transport system, and mobile elements among the strains of phylogroup 2. Russian strains are shown in red. Letters M or D indicate if a strain infects monocots or dicots, respectively. **a** Distribution of the genes of effectors of the type III transport system among the strains of phylogroup 2. Dark blue indicates the presence of a gene, blue indicates partial presence of a gene, and white indicates the absence of a gene. **b** Distribution of mobile elements among the strains of phylogroup 2. Dark blue indicates the presence of a mobile element and white indicates its absence

Using the database on the T3SS effectors in *P. syringae* (http://www.pseudomonas-syringae.org/), we obtained the repertoire of T3SS effectors for each of the studied strains. Fig. 3a shows the distribution of the effectors of the type III secretory system (T3SE) among the strains of phylogroup 2 (Additional file 2). The total number of 81 effectors were found in the strains of phylogroup 2 (Additional file 11): 47 effectors were found in the group M; 78 effectors, in the group D. All the strains have four common effectors: *hopAH1*, *hopAA1*, *hopI1*, and *hopAA1-1*. We checked for the random distribution between groups M and D for each effector using the Fisher’s exact test. Among all of the studied effectors, only *hopBA1* notably distinguishes groups M and D (*p*-value = 0.012). However, it does not pass the correction for multiple testing. Strains 1845 and 2507 have 11 and 10 effectors (Additional file 11), respectively. Five of them are common in these strains: four effectors are common in the entire clade, and the remaining one present in both strains is *hopBA1*.

### Mobile elements, quorum sensing, and CRISPR/Cas-system

The genomes of the second phylogroup were analyzed for the presence of mobile elements (IS). The results are shown in Fig. 3b. The total of 59 elements were found (Additional file 12): 15 of them were common, 14 elements were unique to the group M, and 40 elements were unique to the group D. Moreover, according to Fisher’s exact test, 4 of these 40 elements are significantly more common in the group D, namely ISPsy27, ISPsy28, ISPsy8, and ISPsy6. The presence of ISPs1 significantly determines the group M (*p*-value = 0.02). When correcting for the multiple testing using the fdr method, none of these mobile elements passes by the criterion of significance (*p*-value < 0.05). We found ten IS in strain 1845 and four IS in strain 2507. ISPs1 is present in both groups but none of the strains contain any of the four IS characteristic of the group D strains.

All genomes of the second clade were analyzed for the presence of genes responsible for the quorum sensing system. In gram-negative bacteria of the *Pseudomonas* family, this system is represented either by LuxI-LuxR-genes [44] or by ahlR-ahlI-locus [45]. None of the LuxI system genes were found in the strains of phylogroup 2. The ahlR-ahlI system was found only in several of the D group strains (B728a, CC94, ISPaVe013, ISPaVe037, B301D, and CRAFRU12) that constitute one clade (Fig. 1b). These systems were not found in the Russian strains.

Using the CRISPR-finder service [31], we found that several regions in the Russian strains were identified as the spacer regions of the CRISPR/Cas system. However, the detailed analysis of these regions showed that these are parts of the ice nucleation protein; they also contain repetitions, which the system could mistake for a spacer region. The Cas-9 protein also wasn’t found in strains 1845 and 2507. Apparently, the absence of the CRISPR/Cas system is the feature of *P. syringae* strains [46].

## Discussion

Comparative genomics is often used to find the genes responsible for the virulence and specificity of phytopathogenic bacteria [47]. We compared two previously sequenced groups of phylogroup 2 (clade 2b), infecting monocots and dicots, with two genetically similar *P. syringae* strains of phylogroup 2b; these two strains isolated in Russia in 2000s differ by their specialization against monocots and dicots.

### Comparison of the Russian P. syringae strains

The expansion of crop infection areas and an increase in the harmfulness of *P. syringae* has been reported in Russia since 2004–2007 [48]. The previous epiphytotics of basal bacteriosis and leaf spots in crops (*P. syringae pv. atrofaciens* and *P. syringae pv. syringae*) in Russia was described in the early 1970s [49], which is a bit later than the epiphytotics of similar diseases in 1967–1974 in USA and Canada [50–52], and seminal infection played the major role in the spread of pathogens [50]. Taking into account the earlier emergence of strains SM (isolated in 1990s [53]) and B64 (isolated before 1976 [54–56]), which are the most similar to strains 1845 and 2507, it is possible that the Russian strains have common ancestors with strains B64 and SM. We can assume that the emergence of strains 1845 and 2507 in the Russian Federation is associated with the import of grain from Canada and the United States that took place from the beginning of the 1960s until the 1990s.

To the best of our knowledge, this paper is the first to provide data on sequenced *P. syringae* strains isolated on the territory of the Russian Federation.

The strains isolated in the Russian Federation possess the canonical type III secretion system and a very small set of T3SS effectors. Moreover, the strains contain no functional secretion systems of type IV and VI. We also found no genes of the quorum sensing system, which are crucial to inactivate bactericidal substances synthesized by the host plant. The strains contain a limited number of mobile elements (IS) and have no genes of the CRISPRs system to protect them from bacteriophages and foreign plasmids.

### Comparative phylogenetic analysis

The phylogenetic analysis of strains 1845 and 2507 conducted using the sequence of the *cts* gene fragment [11] and the total of seven household genes (*rpoD, gltA, gap1, gyrB, kup, acnB,* and *pgi*) showed that Russian strains 1845 and 2507 belong to clade 2b of phylogroup 2 of the *P. syringae* species. Moreover, both strains are in the same clade with strains B64 and SM, which also infect monocots [46]. The relationship of these four strains is confirmed by the conservative structure of the hrp cluster, similar ANI values, and high homology of the genomes of strains SM, B64, 1845 and 2507.

Based on the structure of the phylogenetic tree, we can conclude that strain 1845 isolated from a dicotyledonous crop is evolutionarily the youngest and that the change of its host class has occurred recently. We conducted a comparative analysis of strains infecting monocots (including SM, B64, and 2507) and dicots (except 1845) to identify the changes on the genomic level that could lead to the change of the specialization in strain 1845.

### Search for the features of the M and D groups of strains

As expected, the pan- and core genomes of the strain groups M and D (pathogens to monocots and dicots) do not differ at the level of COG-classes (Additional files 9A and 4B). The difference is only observed in unique genes of the genomes of these groups (Additional files 9C and 4D). We also tried to identify associations between the host class and the COG-groups. We identified seven COG-groups (YP_235010.1, YP_234265.1, YP_234264.1, YP_234628.1, YP_235987.1, YP_237386.1, and YP_235613.1) associated with the strains that infect dicots (Table 3, Additional file 13).

Three unique genes (YP_235010.1 YP_237386.1, and YP_235613.1) belong to the LysR group of regulatory proteins. It has previously been shown that the genes of the LysR group play the regulatory role in the expression of the *rovA* gene, which is responsible for the virulence of *Yersinia pseudotuberculosis* enterobacteria [57]. The LysR group genes, *rovM*, are homologous to the virulence regulators PecT/HexA of the *Erwinia* phytopathogens, which is another enterobacterial genus. Unique proteins of the pseudomonas of this group are most similar to the LysR proteins of gram-positive bacteria *Spirosoma linguale* and proteobacteria *Burkholderia ubonensis* and *Brevundimonas diminuta*.

The unique gene YP_234265.1 of the lysin exporter group LysE/YggA also participates in transporting other proteins of bacterial metabolism [58]. Regulatory protein YP_234264.1 of the AsC/Lrp group (leucine-responsive regulatory protein/asparagine synthase C products) is one of the essential bacterial transcription regulators, which determine the metabolism intensity [59]. Interestingly, the groups YP_234264.1 and YP_234265.1 are present in strain 1845, in all strains of the group D, and only in one strain of the group M, namely strain SM, which is the closest to strain 1845 on the phylogenetic tree.

The unique gene YP_234628.1 encodes membrane proteins of the DedA group that participate in the protection of the bacterial membrane in the human and animal pathogens *Salmonella enterica* and *Neisseria meningitides* from cationic cytolytic peptides. These proteins are also necessary for the functioning of the type III secretory system [60]. The unique gene YP_235987.1 encodes the protein of the AraC family of transcription regulators, which control the expression of virulence genes in pathogenic bacteria [61]. The correct functioning of the genes of the third transport system is achieved by the interaction of several regulatory systems affecting the central gene expression regulator of the AraC family [62]. It should be noted that most of the unique genes have the closest homologues outside the *Pseudomonas* genus, in the genomes of pathogenic enterobacteria.

When studying the repertoire of the T3SS effectors, we showed that only 3 out of the total of 81 identified effectors are unique for the group M, whereas the group D contains 37 unique effectors (Additional file 11). However, the average number of the effectors does not differ significantly in the strains of groups M and D (18 ± 8 for the group M and 26 ± 11 for the group D). This fact indicates greater individual variety of the effectors in the strains of the group D, which might partially be explained by the host range. While for the group M hosts came from the same plant family (Poaceae), the range of host organisms of the group D consists of several families.

The analysis of statistical significance of the representation of mobile elements in the strains of groups M and D showed that the mobile element ISPs1 is inherent in the group M, whereas four elements (ISPsy27, ISPsy28, ISPsy8, and ISPsy6) are inherent in the group D. Paper [47] describes ISPs1 as an element unique to the strains that infect monocots [47]. Interestingly, strain 1845 contains ISPs1 but does not contain the four mobile elements characteristic of the group D.

## Conclusions

The genomes of *Pseudomonas syringae* strains 2507 (wheat) and 1845 (sunflower) isolated on the territory of the Russian Federation were determined by pyrosequencing and compared with previously published genome sequences of 18 genomes of the strains belonging to the same phylogroup and affecting dicots and monocots. We analyzed seven informative genes used in MLST genotyping of *P. syringae*, calculated the average nucleotide identity (ANI), studied the synteny of the hrp-gene clusters, and examined the compositions of the type III secretion system (T3SS) effectors and of the elements of insertion sequences (IS). Based on the obtained data, we found that strains 2507 and 1845 and strains SM and B64 (strains SM and B64 were isolated from wheat in the USA in 1990 and before 1976, respectively) form a subgroup that is stable among the other strains of phylogroup 2b. Within this subgroup, the strains 1845 and 2507 demonstrated the greatest similarity in the number of common unique genes. Moreover, the analysis of the genome of strain 1845 indicated the recent loss of several genetic elements (the cluster of genes responsible for the synthesis of syringolin and the prophage cluster) that are present in strains 2507, B64, and SM. We found three genes (YP_234264.1, YP_234265.1, and YP_237386.1), the acquisition of which by strain 1845 could lead to the change in its host class. The obtained results make it possible to perform a detailed study on the role of the identified genes in the specialization of *P. syringae*.

## Additional files

Additional file 1: Results of the artificial inoculation of plants with *Pseudomonas syringae* strains 1845 and 2507 (XLSX 50 kb)
Additional file 2: List of *Pseudomonas syringae* strains selected for phylogenetic analysis (XLSX 57 kb)
Additional file 3: Main morphological, cultural, and biochemical properties of the *Pseudomonas syringae* bacterial strains isolated from sunflower and wheat in different regions of the Russian Federation (XLSX 49 kb)
Additional file 4: Pairwise ANI values for the strains of phylogroup 29 (XLSX 50 kb)
Additional file 5: Pairwise alignment between the genomes of *Pseudomonas syringae* strains (A) SM, (B) 1845, and (C) 2507 produced using MAUVE software (Darling et al. 2010). Colors depict conserved and highly related genomic regions and white areas indicate unique or low identity regions. Blocks shifted below the center line indicate segments that align in the reverse orientation as inversions relative to reference strain SM. (PNG 1177 kb)
Additional file 6: Comparison of the prophage region in *Pseudomonas syringae* strain SM (A) with the corresponding regions in strains 1845 (B) and 2507 (C) using MAUVE software (Darling et al. 2010). (PNG 2118 kb)
Additional file 7: Comparison of the organization of the hrp-gene cluster in *Pseudomonas syringae* strains 2507 (top), 1845 (center), and SM (bottom). Grey arrows indicate genes. Red arrows indicate genes that present in all strains. (PNG 459 kb)
Additional file 8: Pan-genome for the strains of phylogroup 2. Summary table (XLSX 349 kb)
Additional file 9: Distribution of various gene categories (COGs) in different genomes (indicators are presented in percentage): A. Comparison of the COGs distribution in the pan-genomes of groups D and M. B. Comparison of the COGs distribution in the core genomes of groups D and M. C. Comparison of the COGs distribution in unique genes for the pan-genomes of groups D and M. D. Comparison of the COGs distribution in unique genes for the core genomes of groups D and M (PNG 938 kb)
Additional file 10: Results of the COG-class enrichment analysis for the pan- and core genomes of groups M and D (XLSX 50 kb)
Additional file 11: Distribution of effectors of the type III secretory system among the strains of phylogroup 2 (XLSX 274 kb)
Additional file 12: Distribution of mobile elements among the strains of phylogroup 2 (XLSX 50 kb)
Additional file 13: List of seven COG-groups associated with the ability to infect dicots (XLSX 52 kb)
