# Supplementary figures and images for: Comprehensive analysis of draft genomes of two closely related pseudomonas syringae phylogroup 2b strains infecting mono- and dicotyledon host plants
Source: BMC Genomics. 2016 Dec 28;17(Suppl 14):1010. doi: 10.1186/s12864-016-3358-y (PMC5249006; doi:10.1186/s12864-016-3358-y)

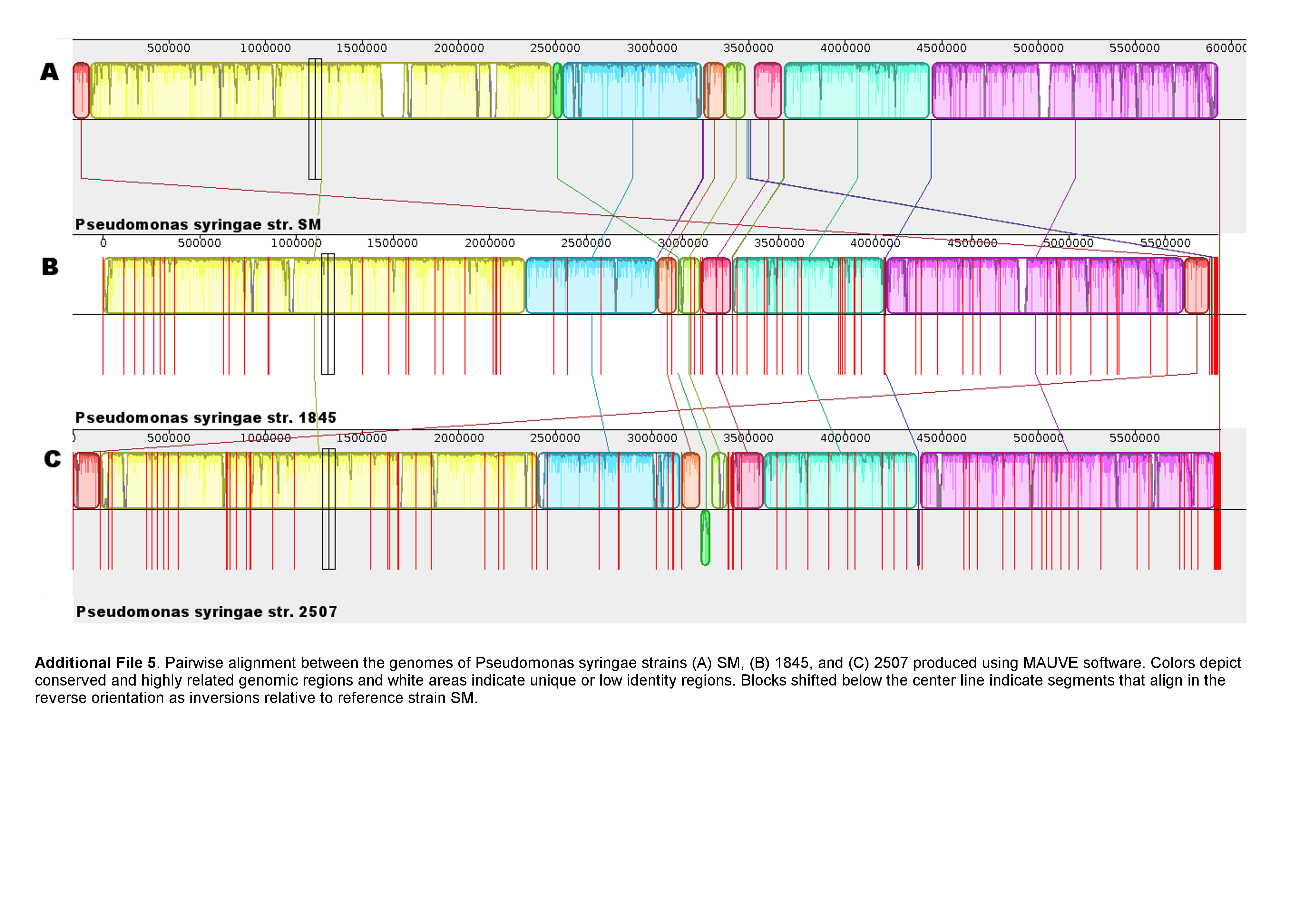

Supplement: Additional file 5: — Pairwise alignment between the genomes of Pseudomonas syringae strains (A) SM, (B) 1845, and (C) 2507 produced using MAUVE software (Darling et al. 2010). Colors depict conserved and highly related genomic regions and white areas indicate unique or low identity regions. Blocks shifted below the center line indicate segments that align in the reverse orientation as inversions relative to reference strain SM. (PNG 1177 kb) [file 12864_2016_3358_MOESM5_ESM.png]

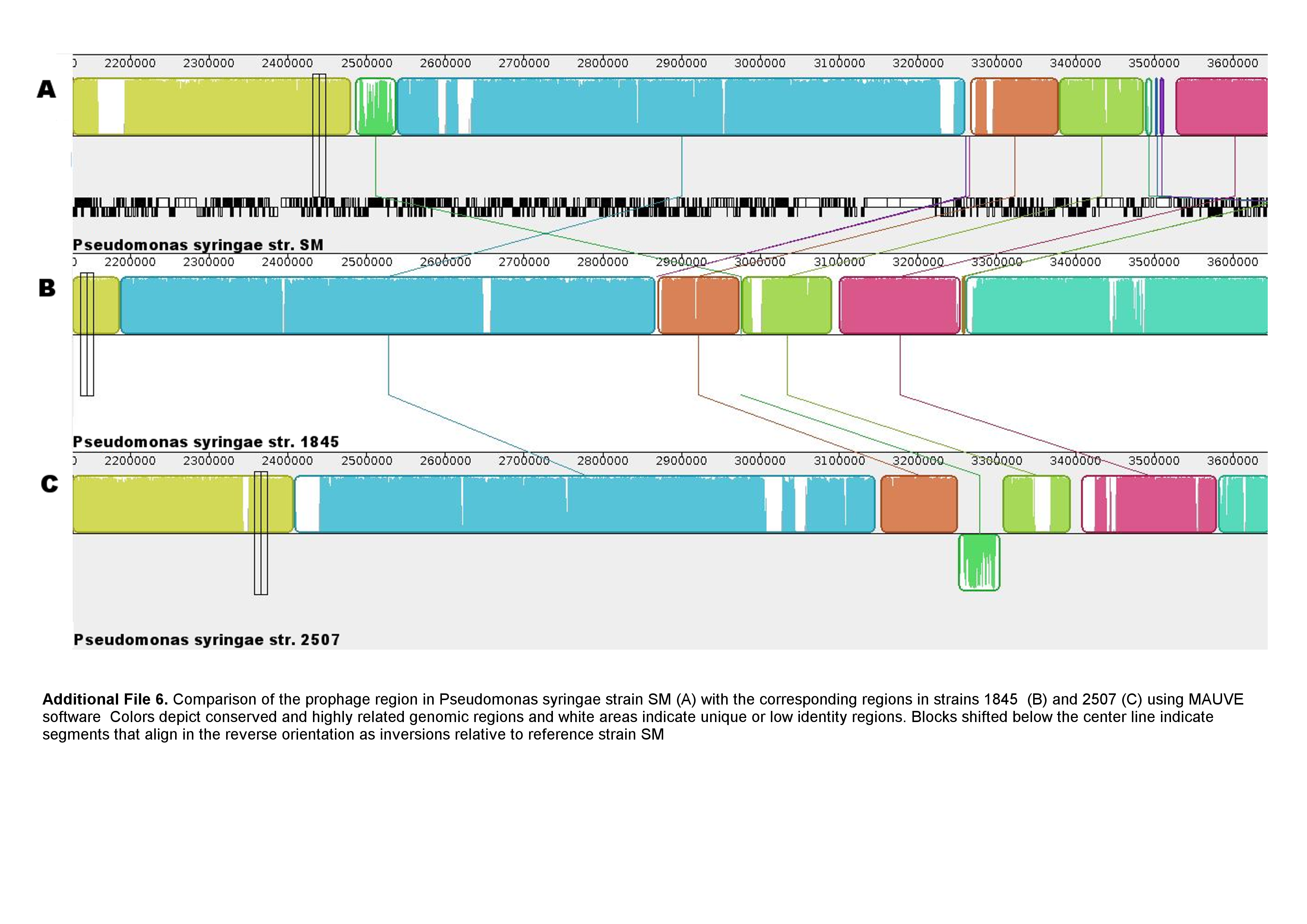

Supplement: Additional file 6: — Comparison of the prophage region in Pseudomonas syringae strain SM (A) with the corresponding regions in strains 1845 (B) and 2507 (C) using MAUVE software (Darling et al. 2010). (PNG 2118 kb) [file 12864_2016_3358_MOESM6_ESM.png]

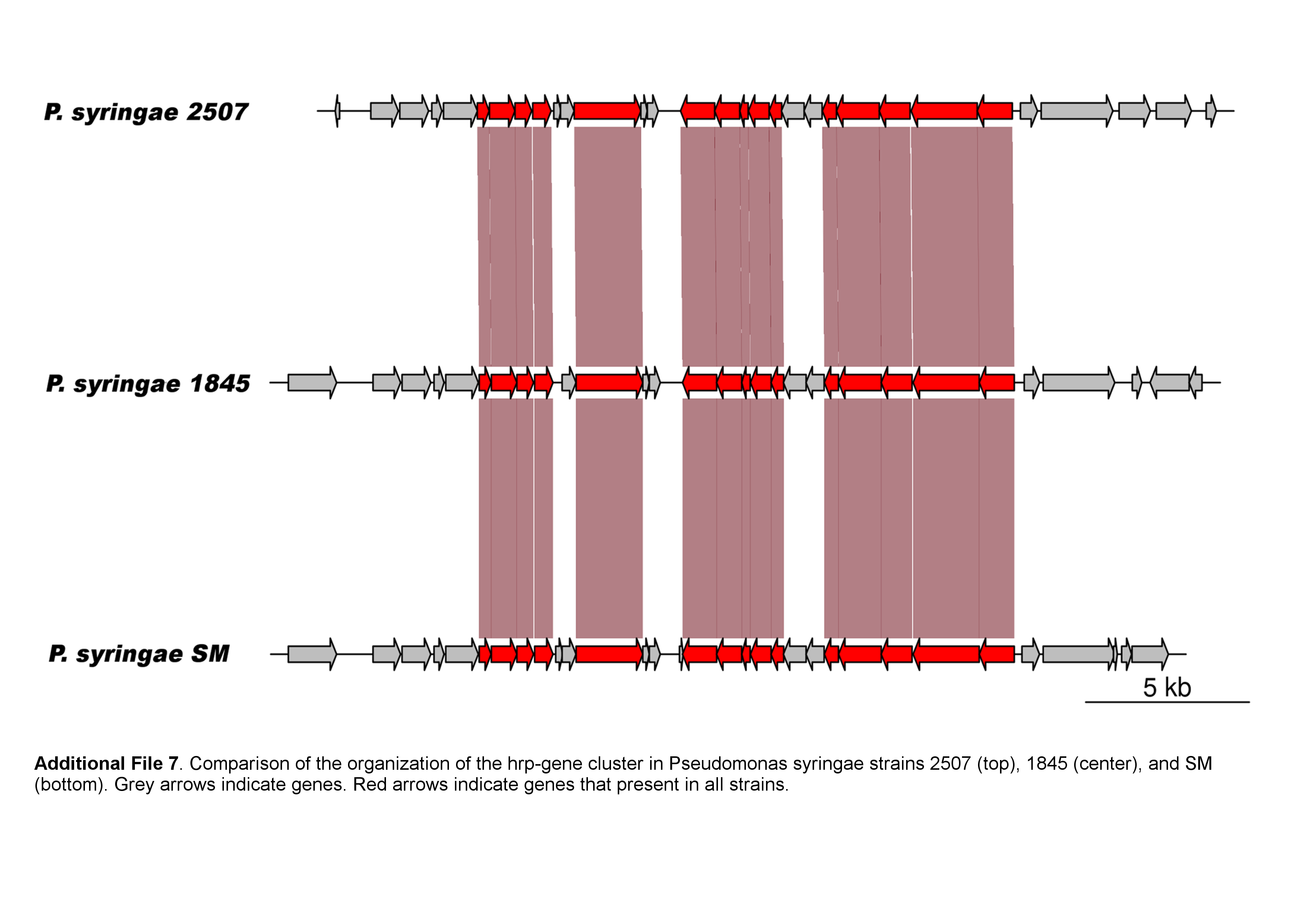

Supplement: Additional file 7: — Comparison of the organization of the hrp-gene cluster in Pseudomonas syringae strains 2507 (top), 1845 (center), and SM (bottom). Grey arrows indicate genes. Red arrows indicate genes that present in all strains. (PNG 459 kb) [file 12864_2016_3358_MOESM7_ESM.png]

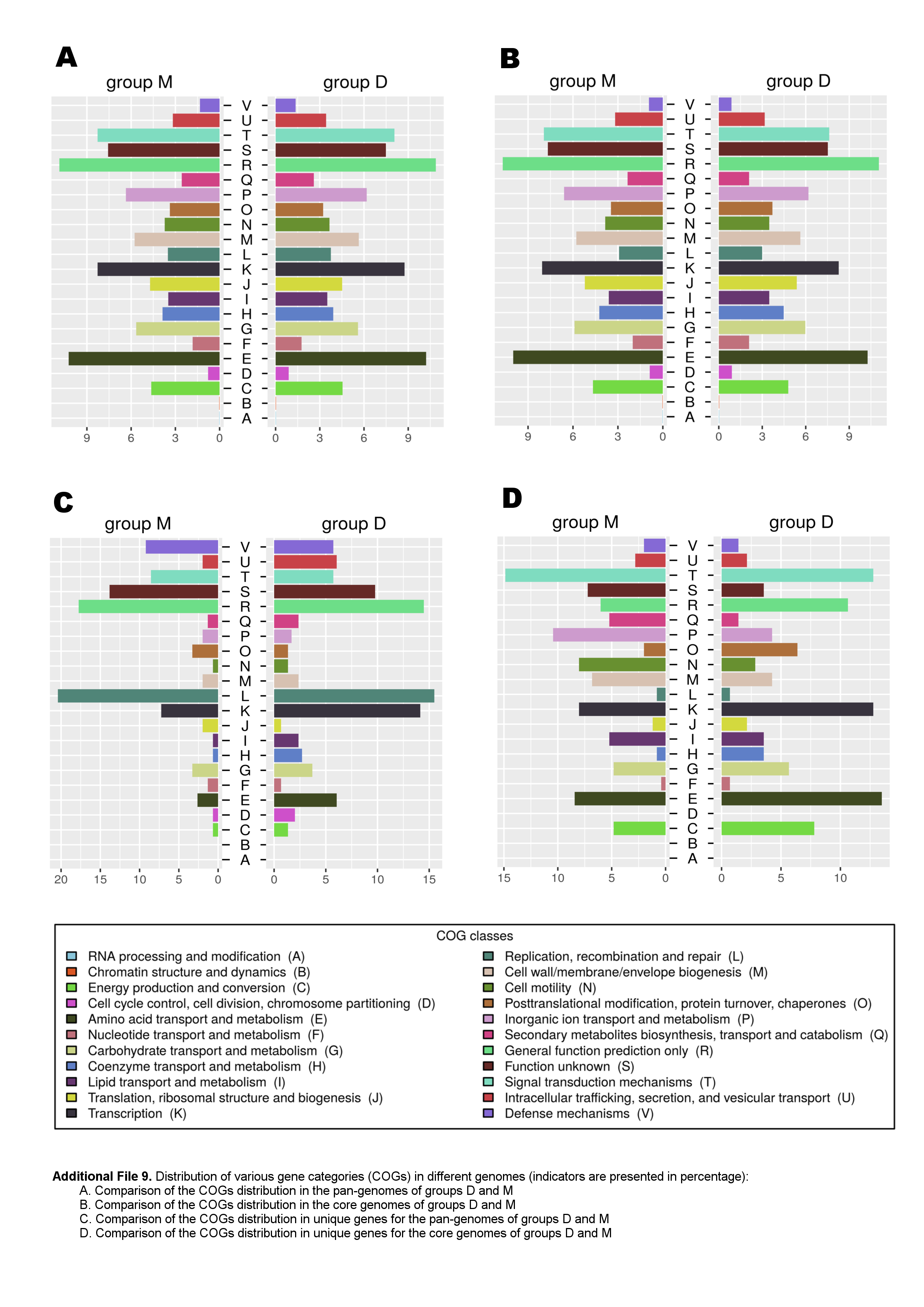

Supplement: Additional file 9: — Distribution of various gene categories (COGs) in different genomes (indicators are presented in percentage): A. Comparison of the COGs distribution in the pan-genomes of groups D and M. B. Comparison of the COGs distribution in the core genomes of groups D and M. C. Comparison of the COGs distribution in unique genes for the pan-genomes of groups D and M. D. Comparison of the COGs distribution in unique genes for the core genomes of groups D and M (PNG 938 kb) [file 12864_2016_3358_MOESM9_ESM.png]
